# Supplementary material for: Geminiviruses encode additional small proteins with specific subcellular localizations and virulence function
Source: Nat Commun. 2021 Jul 13;12:4278. doi: 10.1038/s41467-021-24617-4 (PMC8277811; doi:10.1038/s41467-021-24617-4)
Supplement: Supplementary file 1 — Supplementary Information [file 41467_2021_24617_MOESM1_ESM.pdf]

SUPPLEMENTARY INFORMATION

Geminiviruses encode additional small proteins with specific subcellular localizations and virulence function

Pan Gong[1]*, Huang Tan[2,3]*, Siwen Zhao[1], Hao Li[1], Hui Liu[4], Yu Ma[2,3], Xi Zhang[2,3], Junjie Rong[2,3], Xing Fu[2], Rosa Lozano-Durán[2,5#], Fangfang Li[1#], Xueping Zhou[1,4#]

[1]State Key Laboratory for Biology of Plant Diseases and Insect Pests, Institute of Plant Protection, Chinese Academy of Agricultural Sciences, Beijing, 100193, China [2]Shanghai Center for Plant Stress Biology, CAS Center for Excellence in Molecular Plant Sciences, Chinese Academy of Sciences, Shanghai 201602, China. [3]University of the Chinese Academy of Sciences, Beijing 100049, China. [4]State Key Laboratory of Rice Biology, Institute of Biotechnology, Zhejiang University, Hangzhou, Zhejiang, 310058, China [5]Department of Plant Biochemistry, Centre for Plant Molecular Biology (ZMBP), Eberhard Karls University, D-72076 Tübingen, Germany.

*These authors contributed equally to this work.

[#]Co-corresponding authors: Rosa Lozano-Durán (rosa.lozano-duran@zmbp.uni-tuebingen.de; lozano-duran@psc.ac.cn); Fangfang Li (lifangfang@caas.cn); Xueping Zhou (zzhou@zju.edu.cn).

This file contains:

- **Supplementary Figures 1-12**
- **Supplementary Tables 1-6**

Supplementary Figures

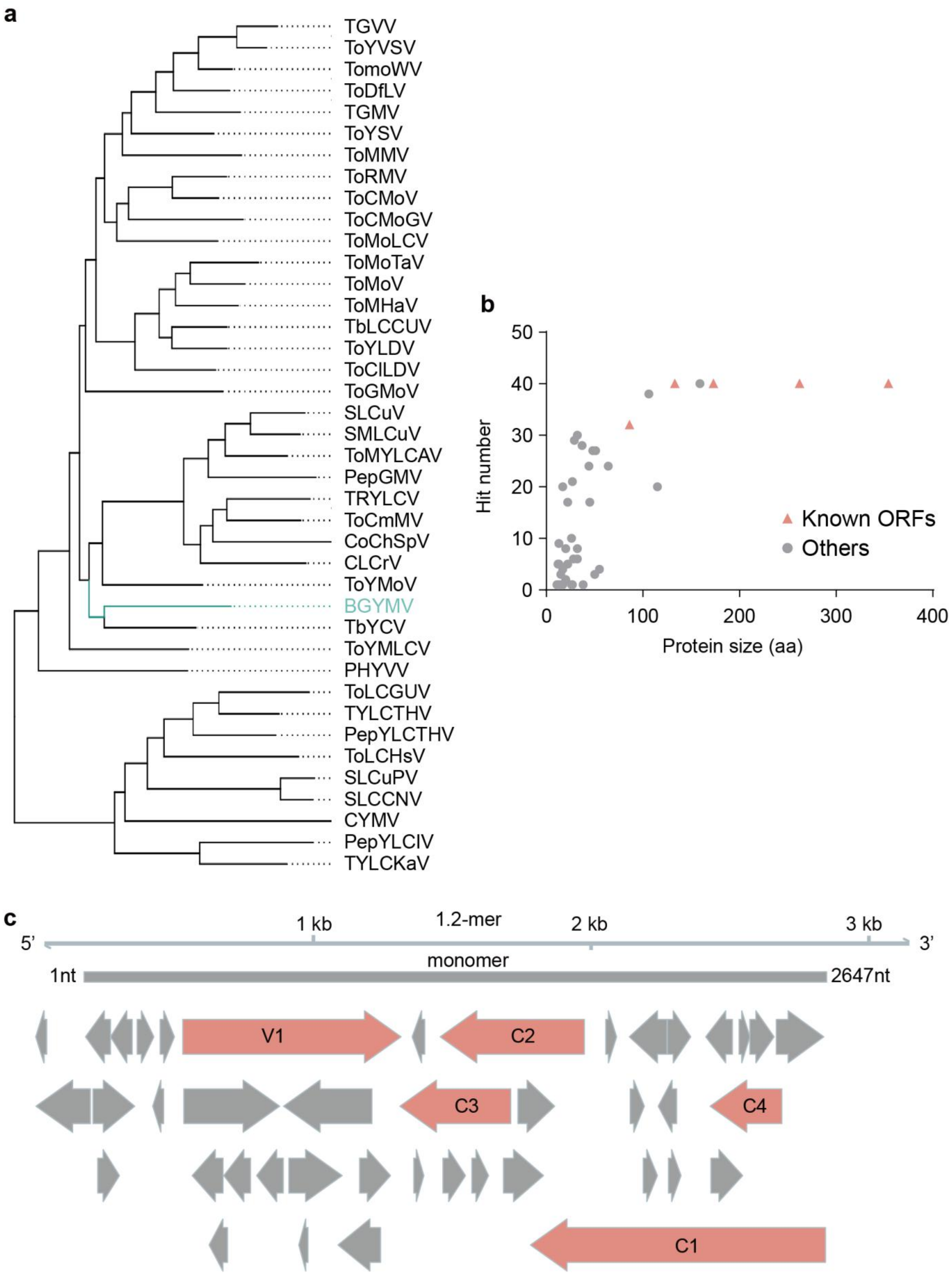

Supplementary Fig. 1. Prediction of ORFs in the bean golden yellow mosaic virus (BGYMV; gen. *Begomovirus*) genome. a. Phylogenic tree based on the full genomic sequences of selected bipartite begomoviruses; for a full list, see Supplementary Table 2. All sequences were downloaded from GenBank. BGYMV is indicated in blue. **b**. Schematic view of predicted ORFs (≥ 30 nt) in the BGYMV genome. A 1.2-mer was used for ORF prediction (see Methods section). **c**. Correlation between the size of the BGYMV ORFs-encoded proteins and their representation in the selected set of bipartite begomoviruses (**a**). In (**b**) and (**c**), arrows indicate ORFs; the known ORFs (C1, C2, C3, C4, and V1) are indicated in pink.

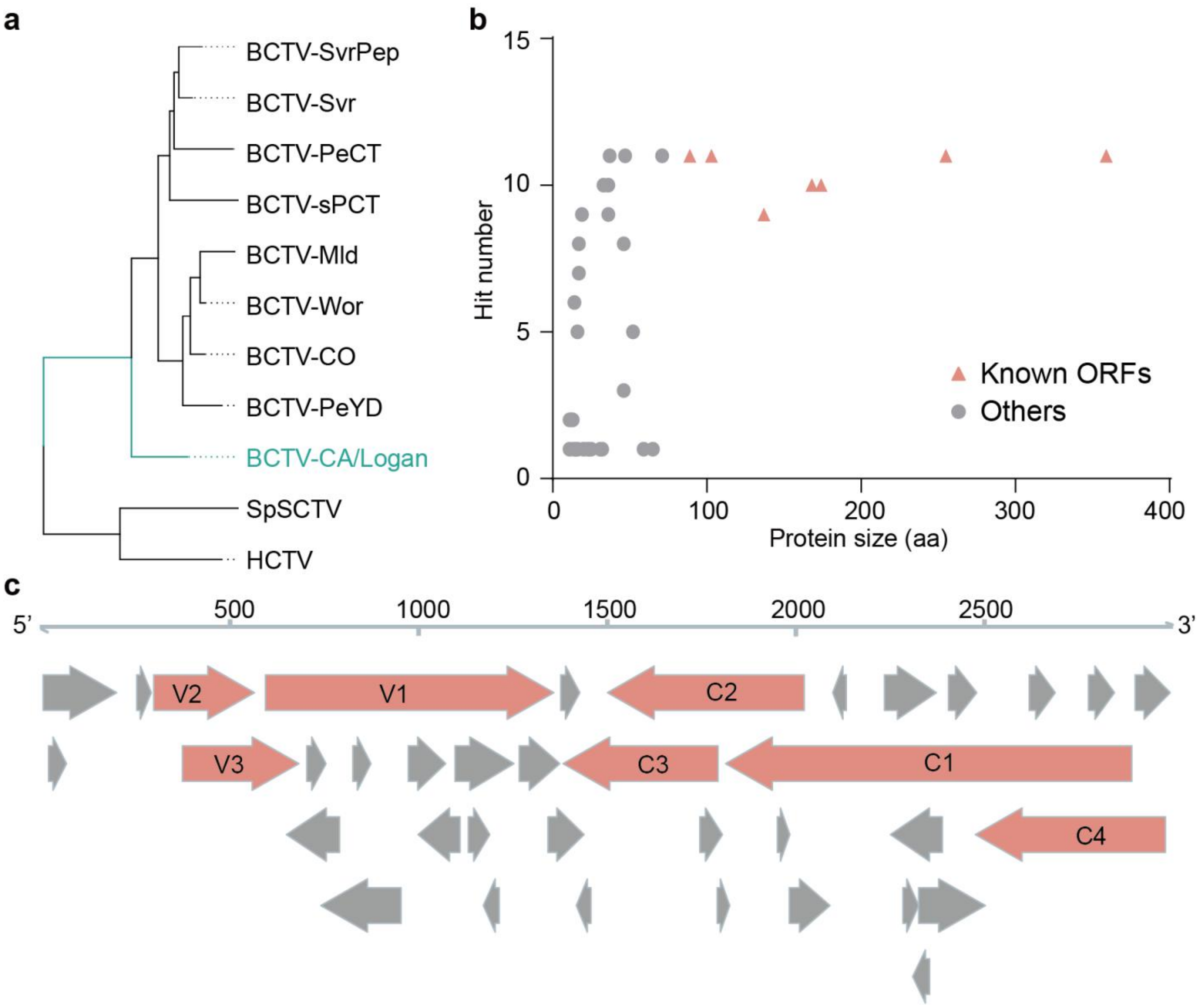

Supplementary Fig. 2. Prediction of ORFs in the beet curly top virus California/Logan strain (BCTV-AC/Logan; gen. *Curtovirus*) genome. a. Phylogenic tree based on full genomic sequences of selected curtoviruses; for a full list, see Supplementary Table 3. All sequences were downloaded from GenBank. BCTV-CA/Logan is indicated in blue. **b**. Schematic view of predicted ORFs (≥ 30 nt) in BCTV-CA/Logan genome. **c**. Correlation between the size of the BCTV-CA/Logan ORFs-encoded proteins and their representation in the selected set of curtoviruses (**a**). In (**b**) and (**c**), arrows indicate ORFs; the known ORFs (C1, C2, C3, C4, V1, V2, and V3) are indicated in pink.

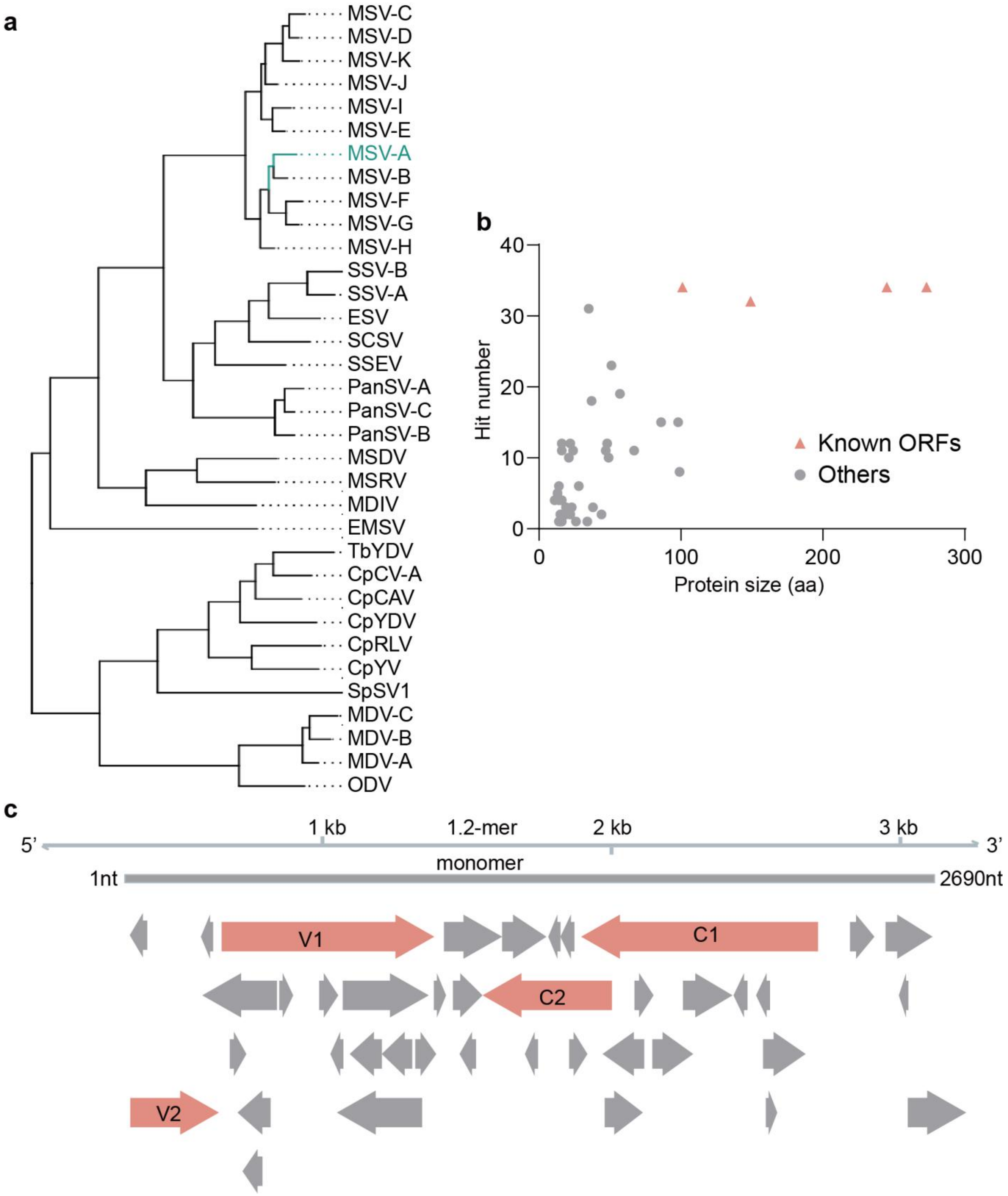

Supplementary Fig. 3. Prediction of ORFs in the maize streak virus A strain (MSV-A; gen. *Mastrevirus*) genome. a. Phylogenic tree based on full genomic sequences of selected mastreviruses; for a full list, see Supplementary Table 4. All sequences were downloaded from GenBank. MSV is indicated in blue. **b**. Schematic view of predicted ORFs (≥ 30 nt) in the MSV-A genome. A 1.2-mer was used for ORF prediction (see Methods section). **c**. Correlation

between the size of the MSV-A ORFs-encoded proteins and their representation in the selected set of mastreviruses (**a**). In (**b**) and (**c**), arrows indicate ORFs; the known ORFs (C1, C2, V1, and V2) are indicated in pink.

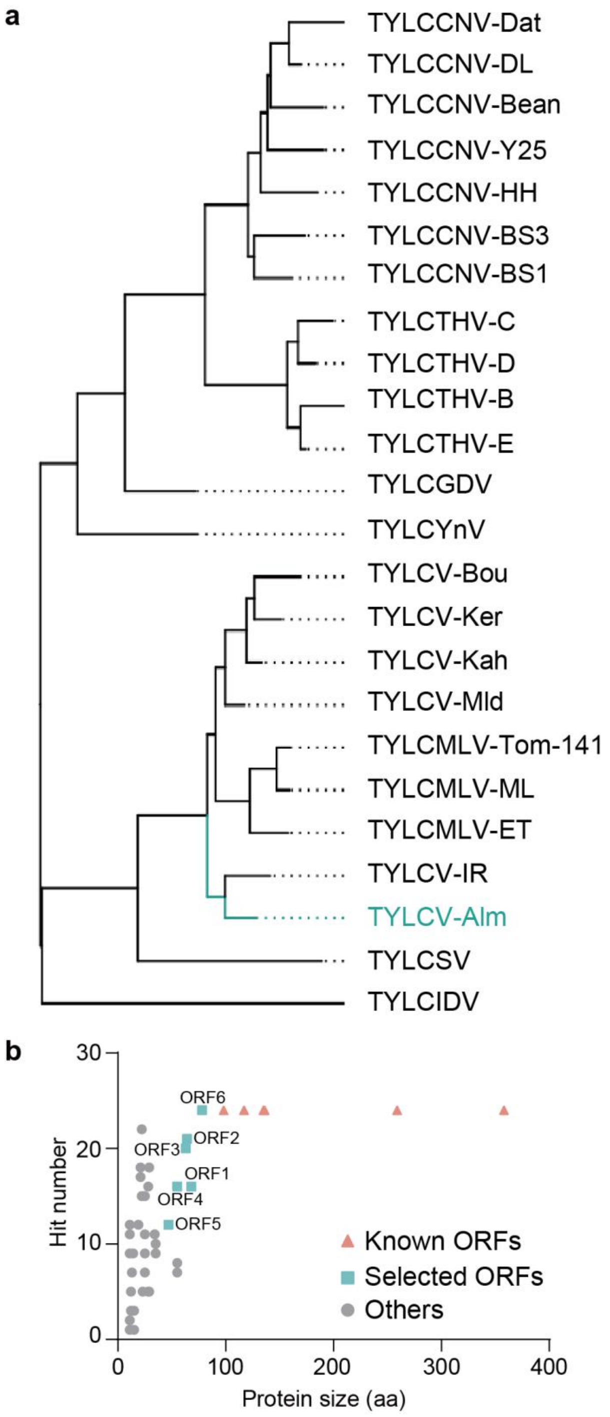

Supplementary Fig. 4. Prediction of ORFs in the tomato yellow leaf curl virus (TYLCV-Alm; gen. *Begomovirus*) genome. a. Phylogenic tree based on full genomic sequences of selected

monopartite tomato-infecting begomoviruses; for a full list, see Supplementary Table 5. All sequences were downloaded from GenBank. TYLCV-Alm is indicated in blue. **b**. Correlation between the size of the TYLCV-Alm ORFs-encoded proteins and their representation in the selected set of monopartite tomato-infecting begomoviruses (**a**). Known ORFs (C1, C2, C3, C4, V1 and V2) are indicated in pink; selected additional ORFs (ORF1-6) are indicated in blue.

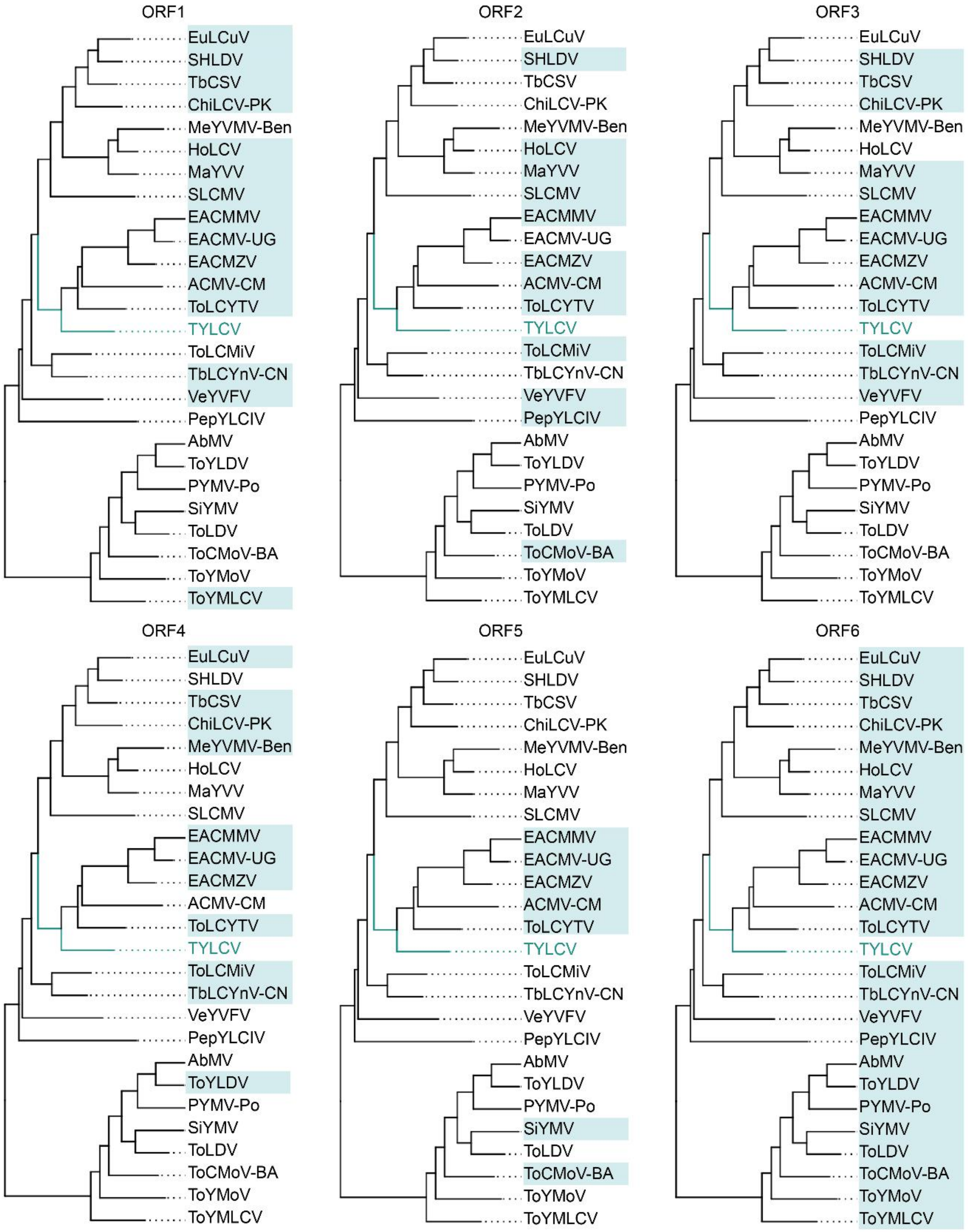

Supplementary Fig. 5. Distribution of ORF1-6 from TYLCV in the selected subset of mono- and bipartite begomoviruses. Blue boxes indicate presence of a given ORF (ORF1-6) described in TYLCV (see Supplementary Fig. 4). For a full list of species, see Supplementary Table 6. All sequences were downloaded from GenBank.

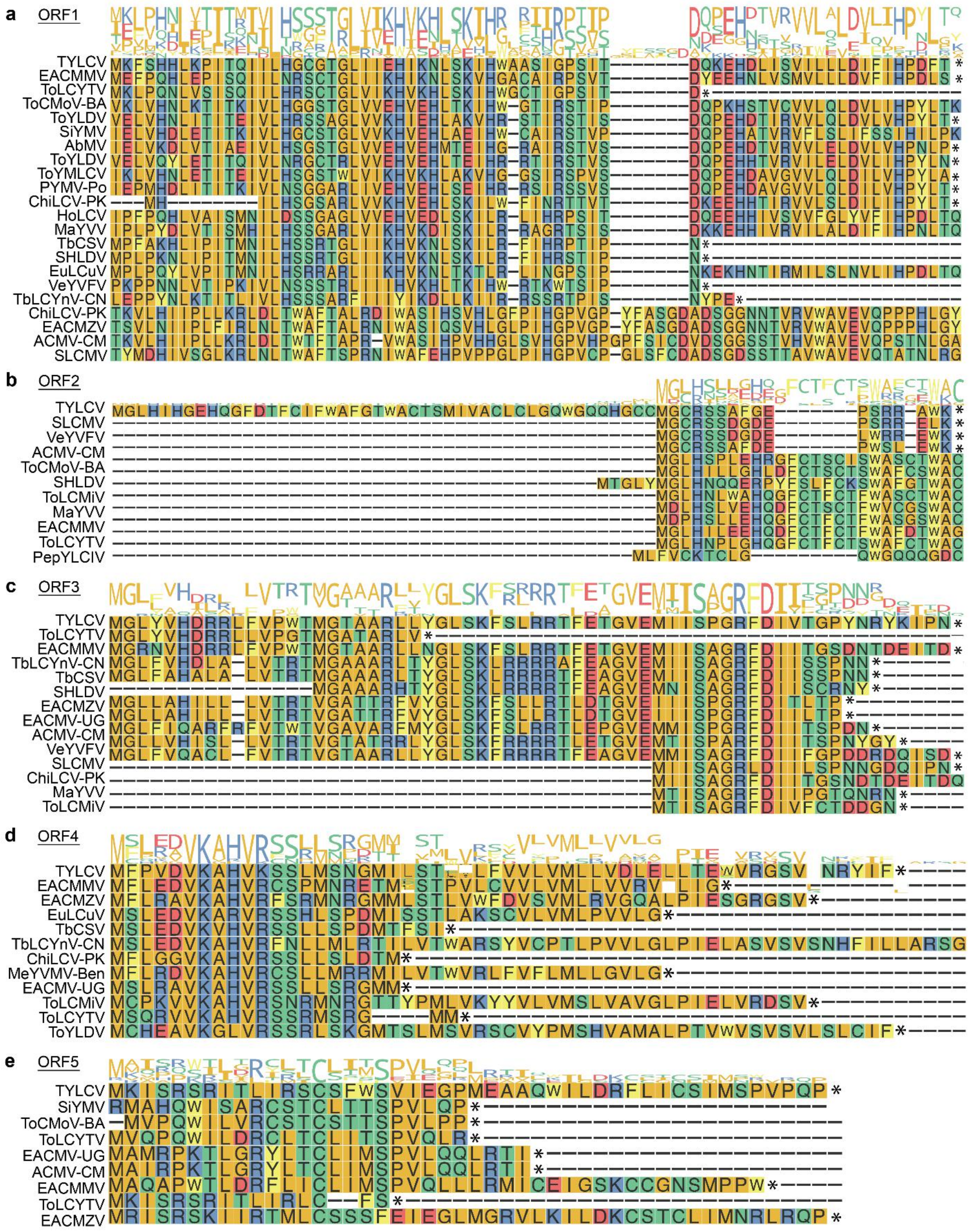

Supplementary Fig. 6. Multiple sequence alignment of the proteins encoded by ORF1-5 in different begomoviruses. Multiple sequence alignment of the proteins encoded by ORF1 (**a**), ORF2 (**b**), ORF3 (**c**), ORF4 (**d**), and ORF5 (**e**). Multiple sequence alignments were performed by ClustalW. Asterisks indicate stop codons.

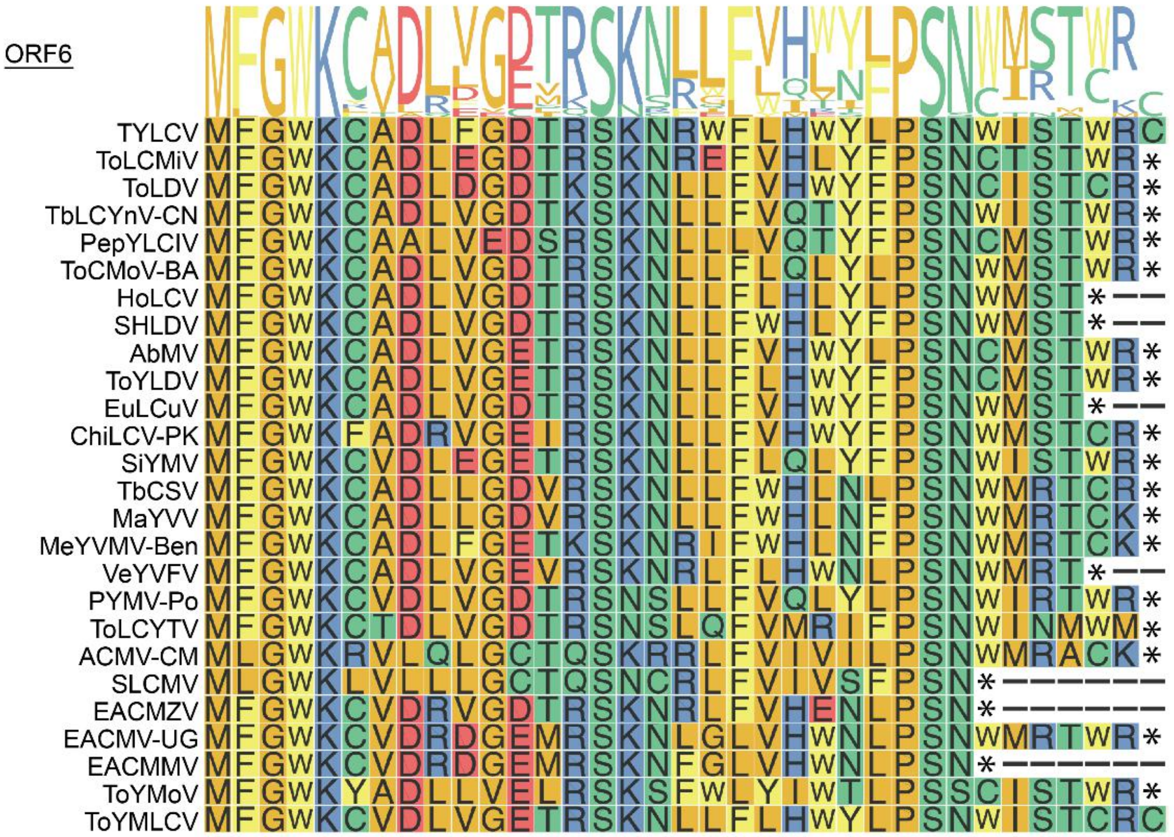

Supplementary Fig. 7. Multiple sequence alignment of the protein encoded by ORF6 in different begomoviruses. Multiple sequence alignments were performed by ClustalW. Asterisks indicate stop codons.

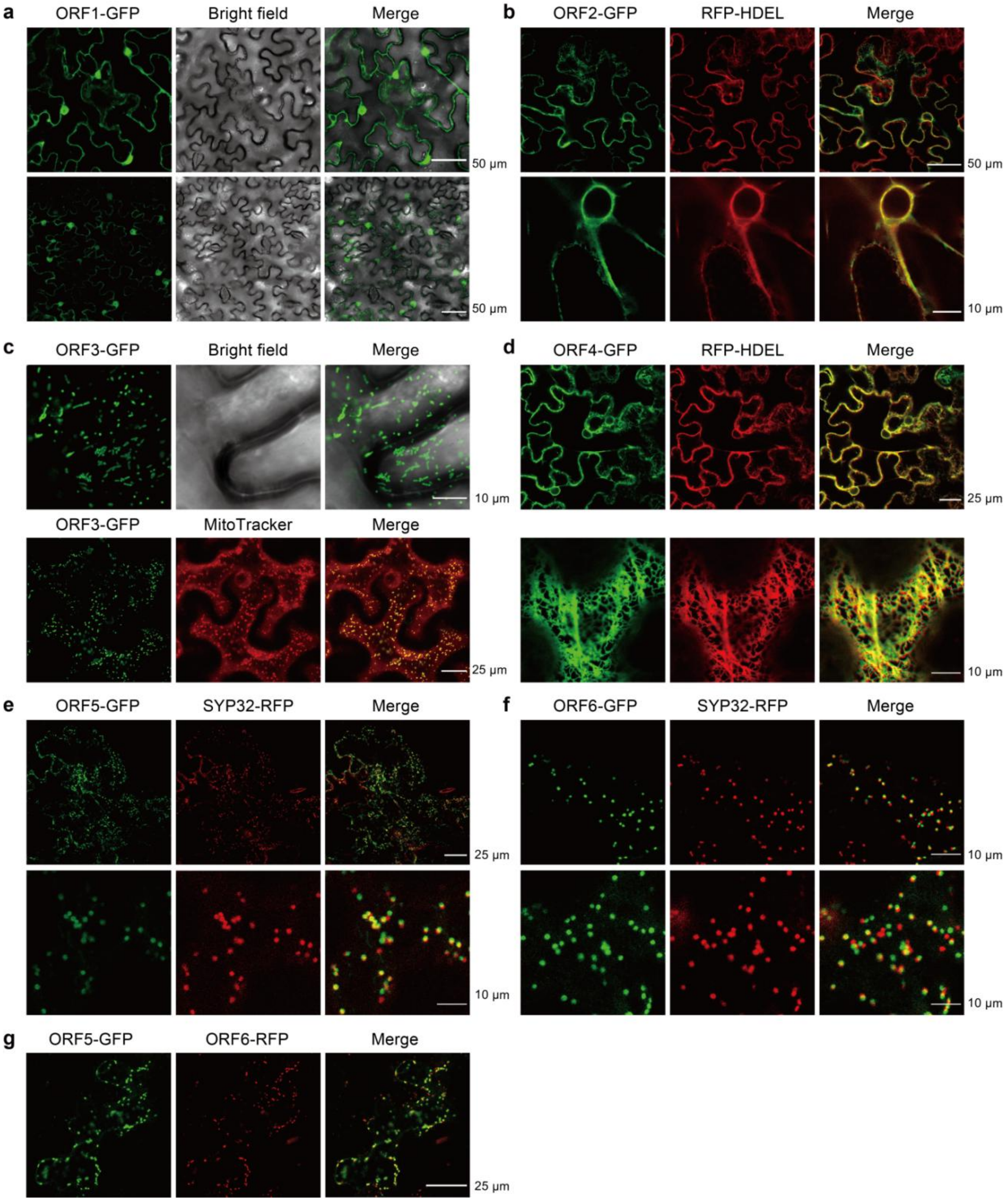

Supplementary Fig. 8. Additional confocal images showing the subcellular localization of GFP-tagged proteins encoded by the selected additional ORFs from TYLCV (ORF1-6) transiently expressed in *N. benthamiana* leaves (related to Fig. 2). RFP-HDEL: ER marker; SYP32-RFP: cis-Golgi marker; MitoTracker Red: mitochondrial staining. The size of scale bars is indicated. These experiments were repeated at least three times with similar results; representative images are shown. The TYLCV isolate used in these experiments is TYLCV-Alm.

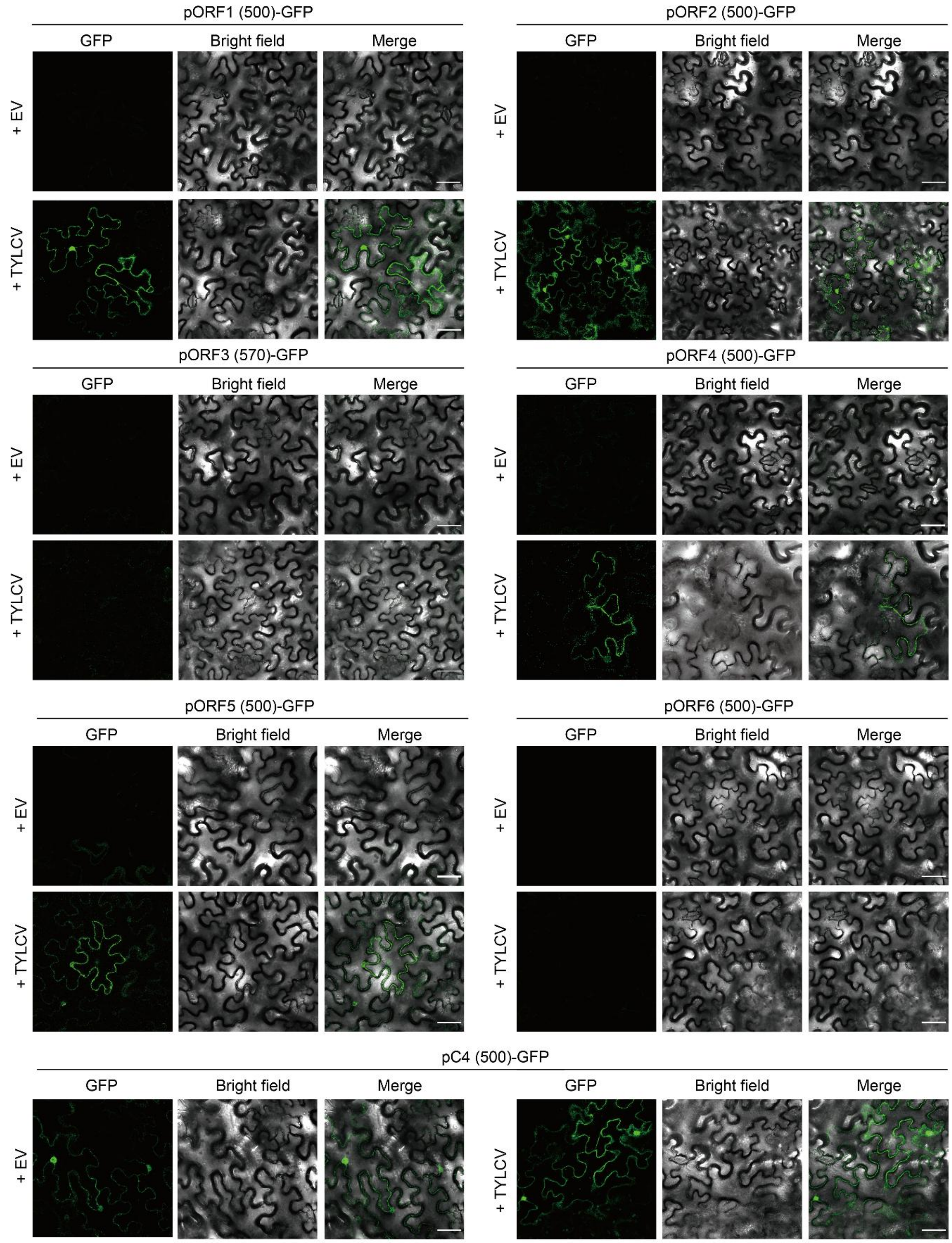

Supplementary Fig. 9. Promoter activity assay of the sequences upstream of ORF1-6. *A. tumefaciens* clones harboring pORF-GFP constructs were used to transiently transform *N.*

benthamiana leaves in the presence (+TYLCV) or absence (+EV) of TYLCV. EV: empty vector. Scale bar: 25 µm. The numbers in brackets indicate the length of the sequenced used, upstream of the ATG of each ORFs, in nt. These experiments were repeated at least three times with similar results; representative images are shown. The TYLCV isolate used in these experiments is TYLCV-Alm.

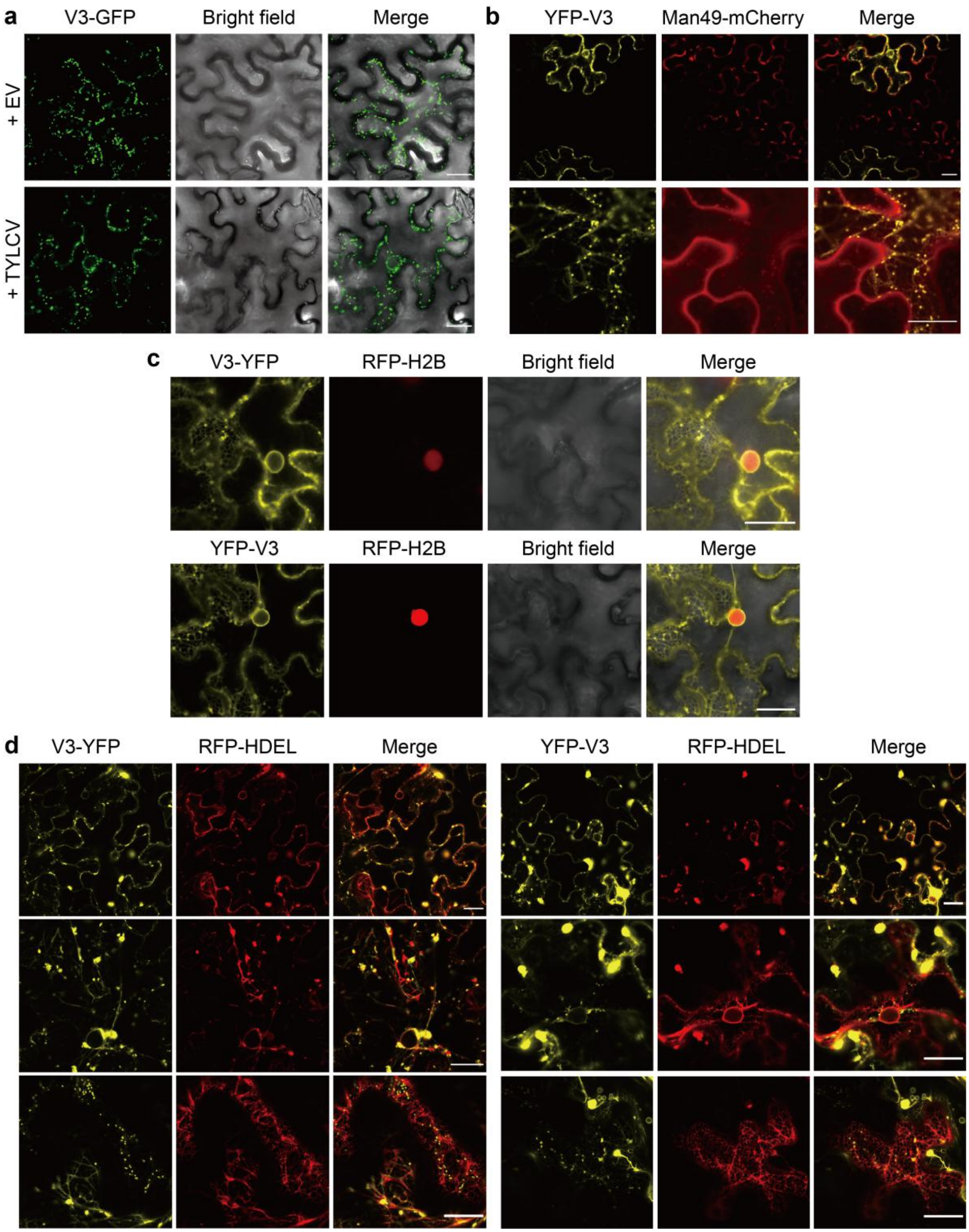

Supplementary Fig. 10. Additional confocal images showing the subcellular localization of V3 with fluorescent protein tags at its N- or C-terminus. a. Subcellular localization of V3-GFP in the presence (+TYLCV) or absence (+EV) of TYLCV. EV: empty vector. Scale bar: 25

µm. **b**. Co-localization of YFP-V3 with the cis-Golgi marker Man49-mCherry. Scale bar: 20 µm. **c**. Subcellular localization of V3-YFP or YFP-V3 transiently expressed in RFP-H2B transgenic *N*. *benthamiana* leaves. Scale bar: 20 µm. **d**. Co-localization of V3-YFP or YFP-V3 with the ER marker RFP-HDEL. Scale bar: 25 µm. **a**-**d**. These experiments were repeated at least three times with similar results; representative images are shown.

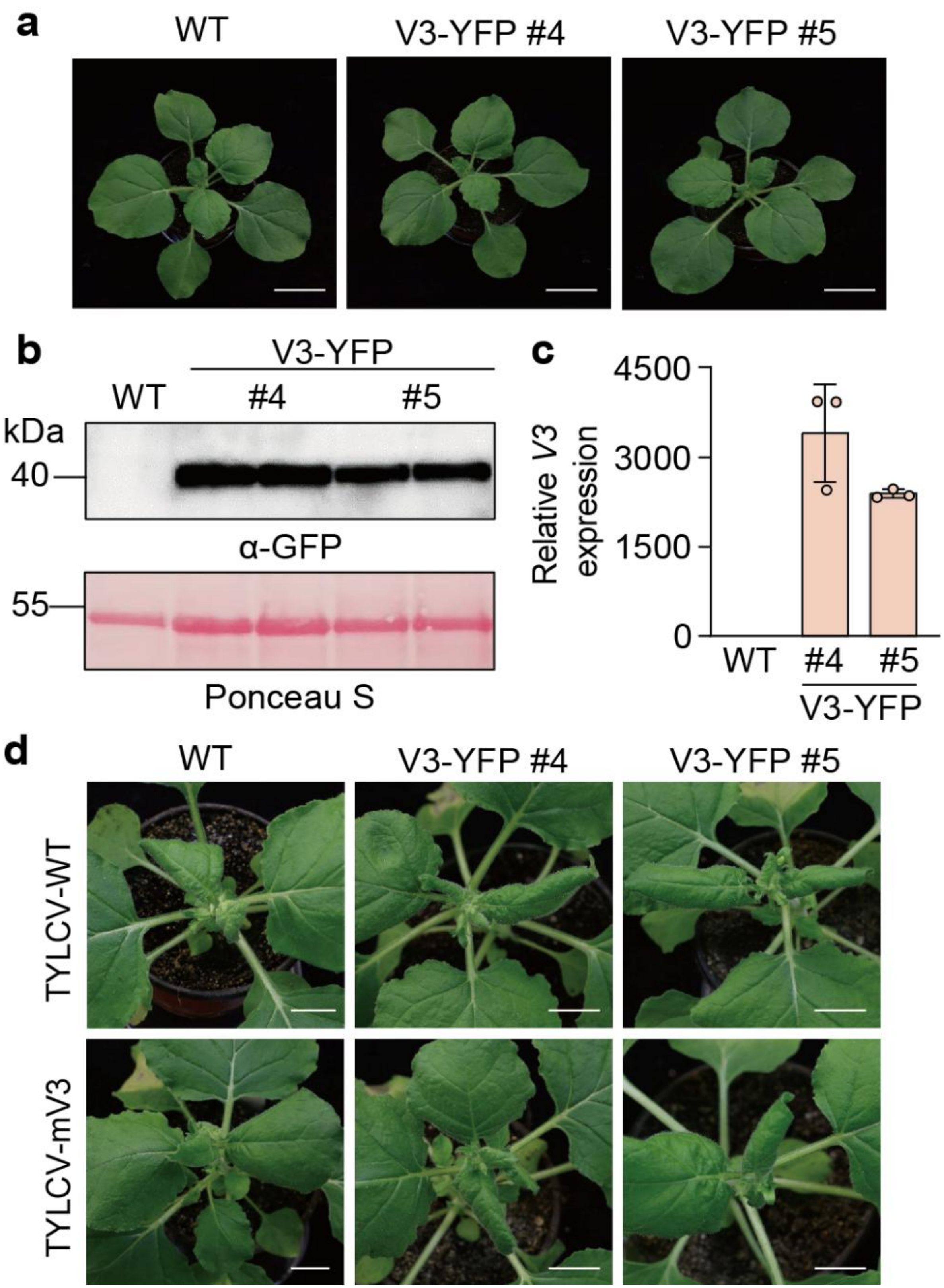

Supplementary Fig. 11. Characterization of transgenic *N. benthamiana* lines expressing V3. a. Phenotype of 5-week-old p35S:V3-YFP T1 transgenic *N. benthamiana* lines (#4 and #5)

compared to the wild type (WT). Scale bar: 2 cm. **b**. Western blot showing V3-YFP protein accumulation in the plants in (**a**). The corresponding Ponceau S staining of the large RuBisCO subunit serves as loading control. This experiment was repeated at least three times independently with similar results. **c**. Relative V3 expression levels in WT and V3-YFP transgenic lines measured by qRT-PCR. Data are the mean of three independent biological replicates. Error bars represent SD. *NbActin2* was used as internal reference. **d**. Symptoms of V3-YFP transgenic *N. benthamiana* plants infected with TYLCV-WT or TYLCV-mV3 at 11 dpi. Scale bar: 2 cm. The original data from all experiments and replicates can be found in the Source data file.

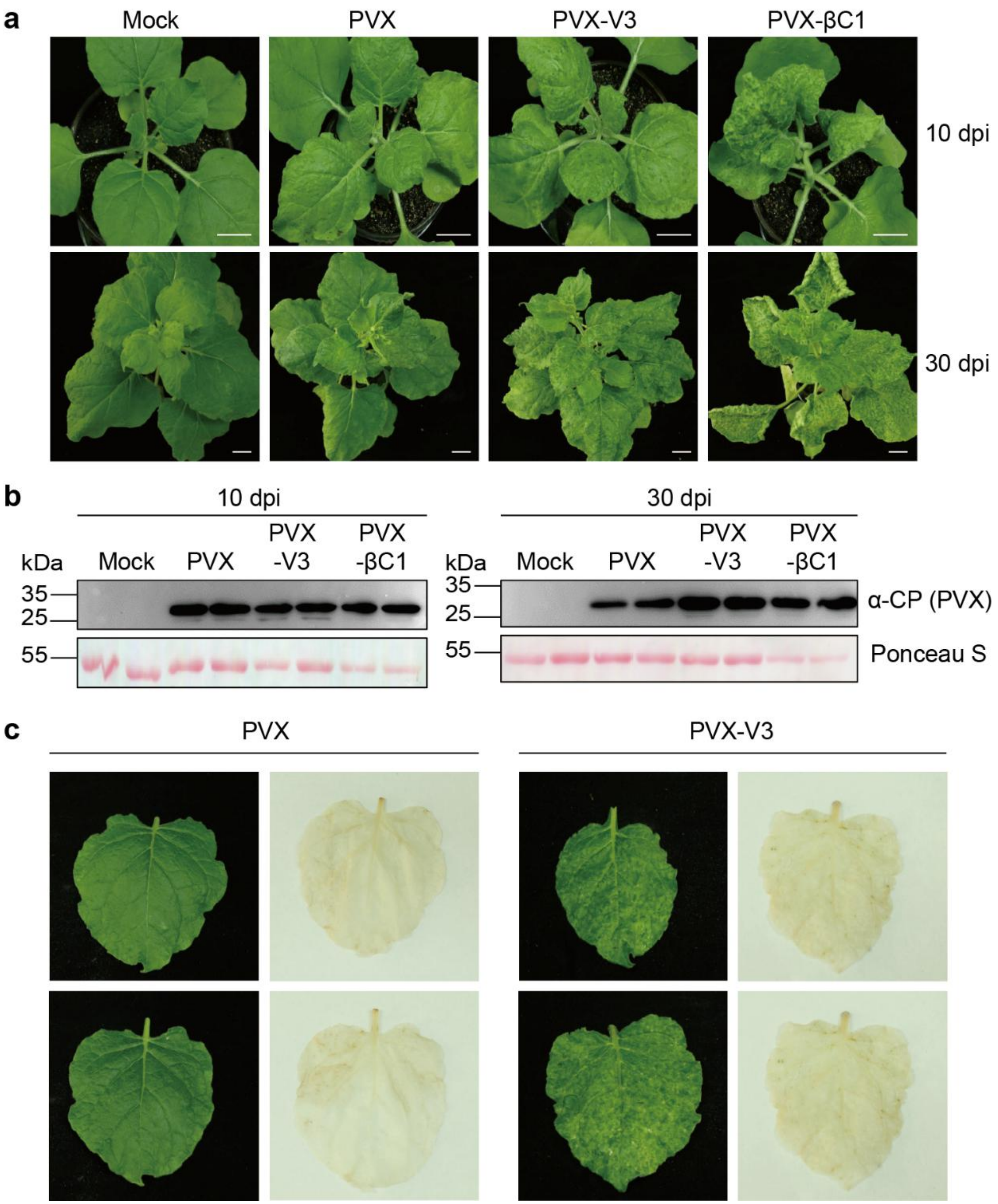

Supplementary Fig. 12. V3 enhances the pathogenicity of PVX. a. Symptoms of *N. benthamiana* plants infected with PVX, PVX-V3, PVX-βC1 (as positive control), or mock-inoculated at 10 dpi (upper panel) or 30 dpi (lower panel). Scale bar: 2 cm. **b**. Western blot showing the accumulation of PVX CP in systemic leaves from (**a**). The corresponding Ponceau

S staining of the large RuBisCO subunit serves as loading control. This experiment was repeated at least three times independently with similar results. **c**. DAB staining of *N. benthamiana* leaves infiltrated with PVX or PVX-V3. The original data from all experiments and replicates can be found in the Source data file.

Supplementary Tables

Supplementary Table 1. Primers used in this work.

Primer name	Sequence (5'-3')	Purpose
ORF1-F	CACCATGGGCCTTCACATCCACGG	To generate TOPO-ORF1-NS
ORF1-NS-R	TTTCCACGCCCGTCTCGAAG	To generate TOPO-ORF1-NS
ORF2-F	CACCATGAAATTTTCTCATCACTTGAAAC	To generate TOPO-ORF2-NS
ORF2-NS-R	GGTAAAGTCTGGATGGATGAA	To generate TOPO-ORF2-NS
ORF3-F	GGGGACAAGTTTGTACAAAAAAGCAGGCTTCATGGGCCTGTACGTCCATGA	To generate pDONR-ORF3-NS
ORF3-NS-R	GGGGACCACTTTGTACAAGAAAGCTGGGTCATTAGGGATCTTATATCTG	To generate pDONR-ORF3-NS
ORF4-F	CACCATGTTCCCCGTGGATGTGAAG	To generate TOPO-ORF4-NS
ORF4-NS-R	AAAAATATATCGATTTAACAC	To generate TOPO-ORF4-NS
ORF5-F	CACCATGAAAATATCAAGAAGCAGA	To generate TOPO-ORF5-NS
ORF5-NS-R	CGGTTGCGGTACTGGGCTCAT	To generate TOPO-ORF5-NS
ORF6-F	CACCATGTTCGGATGGAAATGTGCTG	To generate TOPO-ORF6-NS
ORF6-NS-R	TTTCCTAACATATCCCAATTGT	To generate TOPO-ORF6-NS
SYP32-F	CACCATGTCGGCAAGGCATGGGCAAT	To generate TOPO-SYP32-NS
SYP32-NS-R	TGCCACGAAGAAGAGGAAAATCAT	To generate TOPO-SYP32-NS
ORF1pro500-F	GGGGACAAGTTTGTACAAAAAAGCAGGCTTCAGAGGCATGCGTACATGCCA	To generate pDONR-pORF1(500)
ORF1pro500-R	GGGGACCACTTTGTACAAGAAAGCTGGGTCGTAAAGTCCAGTCTTATGAG	To generate pDONR-pORF1(500)
ORF2pro500-F	GGGGACAAGTTTGTACAAAAAAGCAGGCTTCAACCACGACATCATTTCCATTC	To generate pDONR-pORF2(500)
ORF2pro500-R	GGGGACCACTTTGTACAAGAAAGCTGGGTCGCAACAGTTATTGGTGGGC	To generate pDONR-pORF2(500)
ORF3pro570-F	GGGGACAAGTTTGTACAAAAAAGCAGGCTTCACTGGATTAGAGGCATGCGT	To generate pDONR-pORF3(570)
ORF3pro570-R	GGGGACCACTTTGTACAAGAAAGCTGGGTCCGAAAGCCCAGAATATACAGAATG	To generate pDONR-pORF3(570)
ORF4pro500-F	GGGGACAAGTTTGTACAAAAAAGCAGGCTTCATTACCGGATGGCCGCGCCT	To generate pDONR-pORF4(500)
ORF4pro500-R	GGGGACCACTTTGTACAAGAAAGCTGGGTCCAGGGCTTCGATACATTCTG	To generate pDONR-pORF4(500)
ORF5pro500-F	GGGGACAAGTTTGTACAAAAAAGCAGGCTTCGAATCTGTTCACGGATTTCG	To generate pDONR-pORF5(500)
OR5pro500-R	GGGGACCACTTTGTACAAGAAAGCTGGGTCCCATCCAGACTTTACCTAA	To generate pDONR-pORF5(500)
ORF6pro500-F	GGGGACAAGTTTGTACAAAAAAGCAGGCTTCTGTACCACGCATCATTACTG	To generate pDONR-pORF6(500)
ORF6pro500-R	GGGGACCACTTTGTACAAGAAAGCTGGGTCTCAGGCAGCTAAGAGCTCAAC	To generate pDONR-pORF6(500)
C4pro500-F	GGGGACAAGTTTGTACAAAAAAGCAGGCTTCAGATTCAGGAAATTCA	To generate pDONR-pC4(500)

	TTTAG	
C4pro500-R	GGGGACCACTTTGTACAAGAAAGCTGGGTCTCTCGTGGAGTTCTCTG CAAAC	To generate pDONR-pC4(500)
V3-GSPs-R	GATTACGCCAAGCTTCTCTCTCTAAAGAGGAAGCACTTTCCC	For 5'RACE
V3-nGSPs-R	GATTACGCCAAGCTTGGGGAACCACATCTCCATGTGC	For 5'RACE
q-25S-rRNA-F	ATAACCGCATCAGGTCTCCA	For qPCR
q-25S-rRNA-R	CCGAAGTTACGGATCCATTT	For qPCR
q-TYLCV-F	CCCTCAAAGCTCTATGGCAATCGG	For qPCR
q-TYLCV-R	CAGTGACGTCTGTGGAACCCTC	For qPCR
qV3-F	ATGTTCGGATGGAAATGTGCTGA	For qPCR
qV3-R	GTTTGCAGAGAACTCCACGAGAA	For qPCR and reverse transcription
NbActin2-qPCR-F	AAAGACCAGCTCATCCGTGGAGAA	For qPCR
NbActin2-qPCR-R	TGTGGTTTCATGAATGCCAGCAGC	For qPCR
IF V3pro833 GFP-F	GCTATGACATGATTACGAATTCGCAGCCACAGTCTAGGTC	To generate pCHF3-V3-GFP vector
IF V3pro833 GFP-R	GGATCCCCGGGTACCGAGCTCTCAGGCAGCTAAGAGCTC	To generate pCHF3-V3-GFP vector
IF V3pro833 GUS-F	CCAAGCTGGCGCGCCAAGCTTGCAGCCACAGTCTAGGTC	To generate pINT121-V3-GUS vector
IF V3pro833 GUS-R	GACTGACCTACCCGGGGATCCTCAGGCAGCTAAGAGCTCAAC	To generate pINT121-V3-GUS vector
mV3-F	TTAGCTGCCTGAACGTTCGGATGGAAATG	To generate TYLCV-mV3
mV3-R	CCATCCGAACGTTCAGGCAGCTAAGAGCTCA	To generate TYLCV-mV3
IF-PVX(ClaI)-V3-F	GCACCAGCTAGCATCGAT ATGTTCGGATGGAAATGTG	To generate PVX-V3
IF-PVX(SalI)-V3-R	GTTCATCGGCGGTCGAC TTATTTCCTAACATATCCCAATTG	To generate PVX-V3
221-V3-F	GGGGACAAGTTTGTACAAAAAAGCAGGCTTCATGTTCGGATGGAAAT G	To generate pDONR-V3
221-V3-R	GGGGACCACTTTGTACAAGAAAGCTGGGTCTTTCCTAACATATCCCA ATTG	To generate pDONR-V3

Supplementary Table 2. Selected subset of bipartite begomoviruses (from Supplementary Fig. 1).

**Species**	**Isolate**	**Accession number**	**Virus Abbrev.**
Squash leaf curl Philippines virus	Philippines/Munoz	DNA-A: AB085793; DNA-B: AB085794	SLCuPV
Pepper yellow leaf curl Indonesia virus	Indonesia/2005	DNA-A: AB267834; DNA-B: AB267835	PepYLCIV
Tomato mottle Taino virus	Cuba	DNA-A: AF012300; DNA-B: AF012301	ToMoTaV
Tomato yellow leaf curl Thailand virus	Thailand/2/A	DNA-A: AF141922; DNA-B: AF141897	TYLCTHV/A
Tomato rugose mosaic virus	Brazil/Uberlandia 1/1996	DNA-A: AF291705; DNA-B: AF291706	ToRMV
Squash mild leaf curl virus	United States/Imperial Valley/1979	DNA-A: AF421552; DNA-B: AF421553	SMLCuV
Cotton leaf crumple virus	Mexico/Sonora/1991/Arizona	DNA-A: AF480940; DNA-B: AF480941	CLCrV/AZ
Tomato chlorotic mottle virus	Brazil/Seabra 1/1996/Bahia	DNA-A: AF490004; DNA-B: AF491306	ToCMoV/BA
Squash leaf curl China virus	Vietnam/B/China	DNA-A: AF509743; DNA-B: AF509742	SLCCNV/CN
Tomato yellow leaf curl Kanchanaburi virus	Thailand/Kanchanaburi 1/2001	DNA-A: AF511529; DNA-B: AF511528	TYLCKaV
Tomato leaf curl Gujarat virus	India/Varanasi/2001	DNA-A: AY190290; DNA-B: AY190291	ToLCGUV
Tomato yellow margin leaf curl virus	Venezuela/Merida/57	DNA-A: AY508993; DNA-B: AY508994	ToYMLCV
Tomato mild yellow leaf curl Aragua virus	Venezuela/10/2003	DNA-A: AY927277; DNA-B: EF547938	ToMYLCAV
Tomato yellow spot virus	Brazil/Bicas 2/1999	DNA-A: DQ336350; DNA-B: DQ336351	ToYSV
Tomato golden mottle virus	Mexico/San Luiz Potosi/2005	DNA-A: DQ520943; DNA-B: DQ406674	ToGMoV
Tomato yellow vein streak virus	Brazil/Potato/1983	DNA-A: EF417915; DNA-B: EF417916	ToYVSV
Tomato leaf curl Hsinchu virus	China/Hainan/Ramie/2007	DNA-A: EU596959; DNA-B: EU596960	ToLCHsV
Tomato mild mosaic virus	Brazil/Paty do Alferes 58/2005	DNA-A: EU710752; DNA-B: EU710753	ToMMV
Tomato common mosaic virus	Brazil/Coimbra 22/2007	DNA-A: EU710754; DNA-B: EU710755	ToCmMV
Tomato yellow leaf distortion virus	Cuba/5E17/2007	DNA-A: FJ174698; DNA-B: FJ999999	ToYLDV
Tobacco yellow crinkle virus	Cuba/2007	DNA-A: FJ213931; DNA-B: HQ896204	TbYCV
Tomato chlorotic leaf	Venezuela/Zulia/2004	DNA-A: HQ201952; DNA-B: HQ201953	ToClLDV

distortion virus			
Tomato golden vein virus	Brazil/Ita1220/2003	DNA-A: JF803254; DNA-B: JF803265	TGVV
Tomato rugose yellow leaf curl virus	Uruguay/Salto Grande/U2/2009	DNA-A: JN381819; DNA-B: JN381814	TRYLCV
Tomato dwarf leaf virus	Argentina/Pichanal 397/2008	DNA-A: JN564749; DNA-B: JN564750	ToDfLV
Tomato mottle wrinkle virus	Argentina-Pichanal_400-2008	DNA-A: JQ714137; DNA-B: JQ714138	ToMoWV
Tomato golden mosaic virus	Brazil/Common/1984	DNA-A: K02029; DNA-B: K02030	TGMV
Tomato yellow mottle virus	Costa Rica/2003	DNA-A: KC176780; DNA-B: KC176781	ToYMoV
Tomato mottle leaf curl virus	Brazil/Jaiba 13/2008	DNA-A: KC706615; DNA-B: JF803264	ToMoLCV
Cotton chlorotic spot virus	Brazil/CampinaGrandeB012/2009	DNA-A: KF358470; DNA-B: KF358471	CoChSpV
Tomato chlorotic mottle Guyane virus	French Guyana-Mon2-GF455-2009	DNA-A: KR263181; DNA-B: KR263172	ToCMoGV
Cotton yellow mosaic virus	Benin-Gos_San2-2014	DNA-A: KU683748; DNA-B: KU683750	CYMV
Tobacco leaf curl Cuba virus	Cuba/VC/2015	DNA-A: KX011471; DNA-B: KX011472	TbLCCUV
Pepper yellow leaf curl Thailand virus	Thailand-WF_SPN_Pep-2015	DNA-A: KX943290; DNA-B: KX943291	PepYLCTHV
Tomato mottle virus	United States/Florida/1989	DNA-A: L14460; DNA-B: L14461	ToMoV
Squash leaf curl virus	United States/Imperial Valley/1979	DNA-A: M38183; DNA-B: M38182	SLCuV
Pepper golden mosaic virus	Mexico/Tamaulipas/Tamaulipas	DNA-A: U57457; DNA-B: AF499442	PepGMV/Tam
Pepper huasteco yellow vein virus	Mexico/Tamaulipas	DNA-A: X70418; DNA-B: X70419	PHYVV
Tomato mosaic Havana virus	Cuba/Quivican	DNA-A: Y14874; DNA-B: Y14875	ToMHaV
Bean golden yellow mosaic virus	Dominican Republic/1987	DNA-A: L01635; DNA-B: L01636	BGYMV

Supplementary Table 3. Selected subset of curtoviruses (from Supplementary Fig. 2).

**Species**	**Isolate**	**Accession number**	**Virus Abbrev.**
Beet curly top virus	United States/CA/Logan/1985/California Logan	M24597	BCTV/CA/Logan
Beet curly top virus	Mexico/Mild/2006/Mild	EU193175	BCTV/Mld
Beet curly top virus	United States/Severe/Cfh/Severe	U02311	BCTV/Svr
Beet curly top virus	United States/New Mexico/Severe/Pepper/2001/Severe pepper	FJ545686	BCTV/SvrPep
Beet curly top virus	United States/Colorado/1995/Colorado	JN817383	BCTV/CO
Beet curly top virus	United States/Mild/Worland/Worland	U56975	BCTV/Wor
Beet curly top virus	United States/Spinach 3/1996/Spinach curly top	AY548948	BCTV/ SpCT
Beet curly top virus	United States/New Mexico/Pepper/2005/Pepper curly top	EF501977	BCTV/ PeCT
Beet curly top virus	United States/New Mexico/Pepper/2007/Pepper yellow dwarf	EU921828	BCTV/ PeYD
Horseradish curly top virus	United States/Salinas/1988	U49907	HCTV
Spinach severe curly top virus	United States/Arizona/Spinach 0910/2009	GU734126	SpSCTV

Supplementary Table 4. Selected subset of mastreviruses (from Supplementary Fig. 3).

Species	Isolate	Accession number	Virus Abbrev.
Chickpea chlorosis Australia virus	Australia/2614/2010]	JN989422	CpCAV
Chickpea chlorosis virus	Australia/3455C/2002/A	GU256530	CpCV/A
Chickpea redleaf virus	Australia/Queensland 22/2003	GU256532	CpRLV
Chickpea yellow dwarf virus	Pakistan/PK37/	KM377674	CpYDV
Chickpea yellows virus	Australia/3489B/2002	JN989439	CpYV
Eragrostis minor streak virus	Namibia/Caprivi/g450/2009	JF508490	EMSV
Eragrostis streak virus	Zimbabwe/Guruwe 186/2007	EU244915	ESV
Maize streak dwarfing virus	ET-Adama Zuria-MV1-16	MK329300	MSDV
Maize streak Reunion virus	La Reunion/St Pierre/PR52/2009	JQ624879	MSRV
Maize streak virus	South Africa/A	Y00514	MSV/A
Maize streak virus	South Africa/Worester/Plaas Staal B/g27b/2006/B	EU628597	MSV/B
Maize streak virus	South Africa/Mt Edgecomb/Setaria/1988/C	AF007881	MSV/C
Maize streak virus	South Africa/Rawsonville/1998/D	AF329889	MSV/D
Maize streak virus	South Africa/Mitchelle Park/Natal A/g125/Dig/2006/E	EU628626	MSV/E
Maize streak virus	Nigeria/IITA B/g88/Urochloa/2007/F	EU628629	MSV/F
Maize streak virus	Mali/Mic25/Digitaria/1987/G	EU628631	MSV/G
Maize streak virus	Nigeria/Lagbaka/g79/Setaria/2007/H	EU628638	MSV/H
Maize streak virus	South Africa/New Germany/Natal/A/g217/Digitaria/2007/I	EU628639	MSV/I
Maize streak virus	Zimbabwe/Mic24K/Pennisetum/1987/J	EU628641	MSV/J
Maize streak virus	Uganda/Busia 4/Eustachys/2005/K	EU628643	MSV/K
Oat dwarf virus	Germany/Saxena 25/2006	AM296025	ODV
Panicum streak virus	South Africa/Karino/1989/A	L39638	PanSV/A
Panicum streak virus	Kenya/1990/B	X60168	PanSV/B
Panicum streak virus	Zimbabwe/Guruwe 169/Urochloa/2006/C	EU224264	PanSV/C
Sugarcane chlorotic streak virus	Nigeria/Sc-10/SR/1/2015	KX787914	SCSV
Sugarcane streak Egypt virus	Egypt/Aswan	AF037752	SSEV
Sugarcane streak virus	South Africa/Natal/A	M82918	SSV/A
Sugarcane streak virus	La Reunion/St Pierre/Pie/R5/Cenchrus/2006/B	EU244914	SSV/B
Sweet potato symptomless virus 1	Kenya/Q44429/2005	KY565231	SpSV/1
Tobacco yellow dwarf virus	Australia/1992	M81103	TbYDV
Wheat dwarf India virus	India/2010	JQ361910	WDIV
Wheat dwarf virus	Turkey/barley/A	AJ783960	WDV/A
Wheat dwarf virus	Iran/2008/B	FJ620684	WDV/B
Wheat dwarf virus	Hungary/Kompolt10/1/2010/C	JQ647455	WDV/C

Supplementary Table 5. Selected subset of monopartite begomoviruses causing tomato yellow leaf curl disease (from Supplementary Fig. 4).

Species	Isolate	Accession number	Virus Abbrev.
Tomato yellow leaf curl virus	Spain/Almeria/Pepper/1999	AJ489258	TYLCV
Tomato yellow leaf curl virus	Iran/Genaveh 29/2006/Boushehr	GU076454	TYLCV/Bou
Tomato yellow leaf curl virus	Iran/Iranshahr/1998/Iran	AJ132711	TYLCV/IR
Tomato yellow leaf curl virus	Iran/Kahnooj/2007/Kahnoo	EU635776	TYLCV/Kah
Tomato yellow leaf curl virus	Iran/Hormozgan 32/2006/Kerman	GU076442	TYLCV/Ker
Tomato yellow leaf curl virus	Israel/1993/Mild	X76319	TYLCV/Mld
Tomato yellow leaf curl China virus	China/Guangxi/Honghe	DNA-A: AF311734	TYLCCNV/HH
Tomato yellow leaf curl China virus	China/Yunnan 10/Tobacco/2000/Baoshan	AJ319675	TYLCCNV/BS1
Tomato yellow leaf curl China virus	China/Yunnan 25/Tomato/2000	DNA-A: AJ457985	TYLCCNV
Tomato yellow leaf curl China virus	China/Yunnan 278/Malvastrum/2007/Baoshan3	DNA-A: AM980509	TYLCCNV/BS3
Tomato yellow leaf curl China virus	China/Yunnan/Bean/2004/Bean	DQ256460	TYLCCNV/Bea
Tomato yellow leaf curl China virus	China/Yunnan 5/Tobacco/1999/Dali	DNA-A: AJ319674	TYLCCNV/DL
Tomato yellow leaf curl China virus	China/Yunnan 72/ Datura/2005/Datura	EF011559	TYLCCNV/Dat
Tomato yellow leaf curl Thailand virus	Thailand/Chiang Mai/B	DNA-A: AY514630	TYLCTHV/B
Tomato yellow leaf curl Thailand virus	China/Yunnan 72/2002/C	DNA-A: AJ495812	TYLCTHV/C
Tomato yellow leaf curl Thailand virus	Myanmar/Yangon/1999/D	DNA-A: AF206674	TYLCTHV/D
Tomato yellow leaf curl Thailand virus	Thailand/Sakon Nakhon/E	DNA-A: AY514632	TYLCTHV/E
Tomato yellow leaf curl Guangdong virus	China/Guangzhou 3/2003	DNA-A: AY602166	TYLCGdV
Tomato yellow leaf curl Yunnan virus	China-YN2013-2011	DNA-A: KC686705	TYLCYnV
Tomato yellow leaf curl Mali virus	Burkina Faso/Tom141/2013	LM651400	TYLCMLV
tomato yellow leaf curl Mali virus	Ethiopia/Melkassa/2005/Ethiopia	DNA-A: DQ358913	TYLCMLV/ET
Tomato yellow leaf curl Mali virus	Mali/2003/Mali	AY502934	TYLCMLV/ML
Tomato yellow leaf curl Sardinia virus	Italy/Sardinia/1988	X61153	TYLCSaV
Tomato yellow leaf curl Indonesia virus	Indonesia/Lembang/2005	DNA-A: AF189018	TYLCIDV

Supplementary Table 6. Selected subset of monopartite and bipartite begomoviruses (from Supplementary Fig. 5).

Species	Isolate	Accession number	Virus Abbrev.
Pepper yellow leaf curl Indonesia virus	Indonesia/2005	DNA-A: AB267834; DNA-B: AB267835	PepYLCIV
African cassava mosaic virus	Cameroon	AF112352	ACMV/CM
East African cassava mosaic virus	Uganda/Severe 2/1997/Uganda	DNA-A: AF126806; DNA-B: AF126807	EACMV/UG
Chilli leaf curl virus	Pakistan/Multan/1998/Pakistan	DNA-A: AF336806	ChiLCV/PK
East African cassava mosaic Zanzibar virus	Tanzania/Uguja/1998	DNA-A: AF422174; DNA-B: AF422175	EACMZV
Tomato chlorotic mottle virus	Brazil/Seabra 1/1996/Bahia	DNA-A: AF490004; DNA-B: AF491306	ToCMoV/BA
East African cassava mosaic Malawi virus	Malawi/K/1996	DNA-A: AJ006460	EACMMV
Tobacco curly shoot virus	isolate Y35	AJ420318	TbCSV
Malvastrum yellow vein virus	China/Yunnan 47/2001	AJ457824	MaYVV
Tomato yellow leaf curl virus	Spain/Almeria/Pepper/1999	AJ489258	TYLCV
Tobacco leaf curl Yunnan virus	China/Yunnan 136/2002/China	AJ512761	TbLCYnV/CN
Euphorbia leaf curl virus	China/Guangxi 35/2002	DNA-A: AJ558121	EuLCuV
Sri Lankan cassava mosaic virus	India/Adivaram/2003/India	DNA-A: AJ579307	SLCMV/IN
Tomato leaf curl Mayotte virus	Mayotte/Kahani/2003	AJ865340	ToLCYTV
Sida yellow mosaic virus	Brazil/Vicosa 2/1999	DNA-A: AY090558	SiYMV
Tomato yellow margin leaf curl virus	Venezuela/Merida/57	DNA-A: AY508993; DNA-B: AY508994	ToYMLCV
Potato yellow mosaic virus	Venezuela/1991/Potato	DNA-A: D00940; DNA-B: D00941	PYMV/Po
Mesta yellow vein mosaic virus	Barackpore	EF373060	MeYVMV/Ben
Tomato leaf curl Mindanao virus	Philippines/Mindanao P162/2007	EU487046	ToLCMiV
Tomato leaf distortion virus	Brazil/Paty do Alferes 4/2005	DNA-A: EU710749	ToLDV
Tomato yellow leaf distortion virus	Cuba/5E17/2007	DNA-A: FJ174698; DNA-B: FJ999999	ToYLDV
Sunn hemp leaf distortion virus	India/Barrackpore 1/2008	DNA-A: FJ455449	SHLDV
Hollyhock leaf curl virus	Pakistan/Faisalabad/20/4/06	FR772082	HoLCV
Vernonia yellow vein Fujian virus	CN-Fj-09	JF265670	VeYVFV
Tomato yellow mottle virus	Costa Rica/2003	DNA-A: KC176780; DNA-B: KC176781	ToYMoV
Abutilon mosaic virus	Germany	DNA-A: X15983; DNA-B: X15984	AbMV
